# Effect of an Invasive Plant and Moonlight on Rodent Foraging Behavior in a Coastal Dune Ecosystem

**DOI:** 10.1371/journal.pone.0117903

**Published:** 2015-02-13

**Authors:** Matthew D. Johnson, Yesenia L. De León

**Affiliations:** Department of Wildlife, Humboldt State University, Arcata, California, 95521, United States of America; Texas A&M University at Galveston, UNITED STATES

## Abstract

Understanding how invasive plants may alter predator avoidance behaviors is important for granivorous rodents because their foraging can trigger ripple effects in trophic webs. Previous research has shown that European beach grass *Ammophila arenaria*, an invasive species in coastal California, affects the predation of other seeds by the rodents *Microtus californicus*, *Peromyscus maniculatus*, and *Reithrodontomys megalotis*. This may be due to lower perceived predation risk by rodents foraging in close proximity to the cover provided by *Ammophila*, but this mechanism has not yet been tested. We examined the perceived predation risk of rodents by measuring the ‘giving up density’ of food left behind in experimental patches of food in areas with and without abundant cover from *Ammophila* and under varying amount of moonlight. We found strong evidence that giving up density was lower in the thick uniform vegetation on *Ammophila*-dominated habitat than it was in the more sparsely and diversely vegetated restored habitat. There was also evidence that moonlight affected giving up density and that it mediated the effects of habitat, although with our design we were unable to distinguish the effects of lunar illumination and moon phase. Our findings illustrate that foraging rodents, well known to be risk-averse during moonlit nights, are also affected by the presence of an invasive plant. This result has implications for granivory and perhaps plant demography in invaded and restored coastal habitats. Future research in this system should work to unravel the complex trophic links formed by a non-native invasive plant (i.e., *Ammophila*) providing cover favored by native rodents, which likely forage on and potentially limit the recruitment of native and non-native plants, some of which have ecosystem consequences of their own.

## Introduction

As plant invasions continue to alter ecosystems worldwide [1], [2], it becomes increasingly important to understand their effects on trophic relationships of native species. Invasive plants can dramatically alter the structure and composition of areas they invade [3], [4], effectively creating novel habitats [5]. Habitat composition and structure are well known to affect animal behavior [6], including behaviors to minimize predation risk [7], but to date there have been few studies examining how plant invasions may affect consumers’ avoidance of predation [8].

Understanding how invasive plants may alter predator avoidance behaviors is especially important for granivorous rodents because their foraging can trigger ripple effects in trophic webs [9], [10]. If invasive plants provide a novel high-cover and low-risk habitat for native consumers, the resulting shifts in foraging behavior can alter the impact of consumers on plant populations [11], [12]. Foraging animals are also affected by abiotic factors such as temperature [13], rainfall [14] and, especially for nocturnal mammals, moonlight [15], [16]. However, we are aware of only one previous study that assessed how an abiotic factor (moon phase) may act in concert and possibly interact with effects of invasive plant species on animal foraging. Mattos and Orrock [8] found that the exotic invasive shrub *Lonicera maackii* decreased perceived predation risk for rodents, but this effect was contingent on both moonlight and temperature.

The invasive European beach grass *Ammophila arenaria* (hereafter, *Ammophila*) was planted in California USA in the early 1900s to stabilize coastal dune habitats. It is now fully naturalized in central and northern California and has supplanted populations of native dune plants in many areas, including the North Spit of Humboldt Bay in northwestern California [17]. It reaches notoriously high levels of cover that differ drastically from sparser native dune plant communities, effectively creating a novel habitat in both composition and structure [18]. Maron and Simms found rodent granivory can strongly affect plant demography in California’s coastal ecosystems ([19, [20]), and the novel high cover provided by *Ammophila* can affect rodent foraging and its consequences. For example, Dangremond et al. [21] showed that, when close to *Ammophila*-dominated habitat, the endangered coastal dune plant *Lupinus tidestromii* experiences high levels of predispersal seed consumption by rodents. This may be due to lower perceived predation risk by rodents foraging in close proximity to the cover provided by *Ammophila*, but this mechanism has not yet been tested.

Foraging habitat selection by rodents can be measured by the use of ‘giving-up-densities’ (GUDs; e.g., [7], [22], [23]). In a depletable food patch, harvest rate for a consumer diminishes as food becomes increasingly scarce. The GUD is the density of food at which a forager ceases to feed in a food patch, which should occur when the marginal benefit of harvesting no longer outweighs the marginal cost of foraging. Foraging costs include metabolism, cost of harvesting, predation risk, and the cost of missed opportunities, i.e., the cost of not foraging elsewhere [7]. Thus, the GUD reflects the harvest rate when a rodent decides to stop foraging [22]. Trays containing seeds mixed with local sand have been used extensively to assess GUDs and measure how granivores value different foraging patches, especially in arid environments [24]. However, there has been very little research on GUDs for rodents in coastal ecosystems [13], despite the potential for rodent foraging to strongly affect coastal ecosystem processes [19].

Here, we examined rodent foraging in a coastal ecosystem by measuring GUDs in areas with and without abundant cover from *Ammophila* and under varying amount of moonlight. We hypothesized that perceived predation risk would be lower (i.e., lower GUD) in the *Ammophila*-dominated habitat because of abundant cover [23]. We also hypothesized that if moonlight helps predators capture their rodent prey more than it helps rodents evade predators [25], [26], [27], then rodent perceived predation risk (and GUD) should increase with increasing moonlight. Finally, we hypothesized that the perceived safety provided by cover may depend on moonlight, with strongest differences during nights with more lunar light.

## Materials and Methods

**Fig 1:**
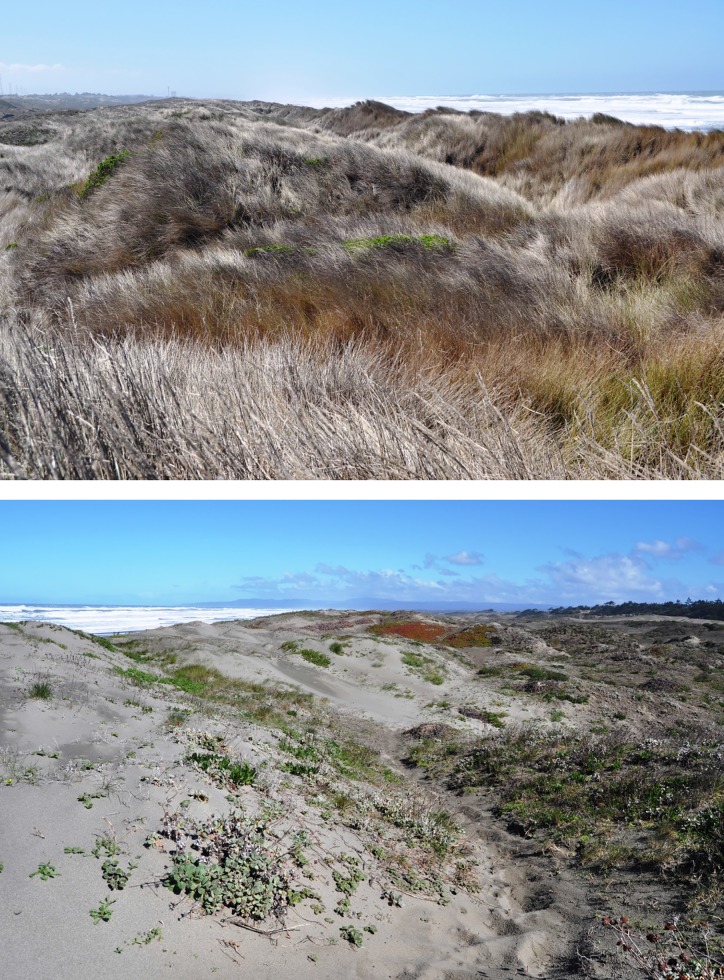
Invaded and restored habitat near Humboldt Bay, California USA. Habitat invaded and dominated by European beach grass, *Ammophila arenaria* (top), and restored habitat with higher plant diversity and lower vegetative cover (bottom). Photographs by M. Johnson.

This study was conducted June-August 2011 on three pairs of sites in two coastal dune habitats on the North Spit of Humboldt Bay in northwestern California. The specific locations of the studies avoided protected species (40°51’18.0”N, 124°09’53.1”W; 40°51’33.3”N, 124°09’51.3”W; 40°51’38.2”N, 124°09’58.2”W). One habitat was dominated by *Ammophila*, whereas the other habitat was restored and free of *Ammophila* (Fig. 1). Our *Ammophila*-dominated sites were located on a private 46-ha reserve owned and managed by a nongovernmental organization, Friends of the Dunes. On these sites, the plant community was comprised almost entirely of *Ammophila*. These *Ammophila* stands are notoriously dense, and canopy cover ranged from 70–100% (see Results for more details of plant community). Our restored sites were located on adjacent land on the southern portion of Ma-le’l Dunes Cooperative Management Area, a 62-ha parcel owned and managed by the Bureau of Land Management (BLM), who granted permission for the field research. The BLM partnered with the California Conservation Corps (CCC) for 14 years to achieve complete removal of *Ammophila* in the foredunes, thus making way for ongoing native American dunegrass (Leymus mollis ssp. mollis) recovery efforts [28]. Thus, this habitat is a restored coastal dune site dominated by native plant species that are maintained naturally at much lower levels of cover than in the *Ammophila*-dominated habitat. Canopy cover ranged from 10–80% (see Results for more details of the plant community). Permission for our field studies was obtained directly from Friends of the Dunes and the Bureau of Land Management (Arcata office), who should be contacted for future research permissions.

We examined perceived predation risk by measuring GUDs on three locations with two sites each, one in *Ammophila*-dominated habitat and the other in restored habitat. In each site, GUD was measured from 25 food stations arranged in a 5×5 grid (5 m spacing between stations) placed in each site. Each food station contained an uncovered clear plastic tub (Rubbermaid #3Q24; 42.7 × 33.8 × 13.5 cm) with six holes (7.6 cm diameter) drilled on the sides to allow for rodent accessibility and filled with 10.0 g of shelled sunflower seeds (kernels) mixed in with 1.0 L of sifted local dune sand. Livetrapping indicates the dominant rodents in this system are California voles (*Microtus californicus*), deer mice (*Peromyscus maniculatus*), and western harvest mice (*Reithrodontomys megalotis;* unpublished data). These rodents are primarily granivorous and nocturnal during the dry summer months in California [29], [30]. To collect nocturnal GUD data, sunflower seeds were deployed at dusk (19:00–21:00 PDT) and those remaining the following morning were sifted through wire mesh and collected. Patches were refilled with 1 L of sand and 10.0 g of fresh sunflower seeds. In the laboratory, collected seeds were separated from other particles, dried in an oven (60°C for at least 56 h) and then weighed. The mass in grams of the dried seeds is the GUD for a given patch on a given night. This process was repeated for six nights on each grid. To avoid confounding temporal effects with habitat comparisons, we ran the two grids at each location simultaneously, one on an *Ammophila*-dominated site and one on a restored site. Location 1 was run from 19 to 24 June, location 2 was run 5–10 July, and location 3 was run 20–25 July.

Rodents are known to respond to lunar cycle independent of visual stimuli, with behavioral activity tracking moon phase [31], [32]. For each night, we obtained moon phase data for our area from the US Naval Observatory’s sun and moon data portal [33], reported as the fraction of the moon illuminated during each night in our study on a continuous scale of 0 (new moon) to 1.0 (full moon). However, on-the-ground visual lunar illumination is also affected by the rise and set times of the sun and moon, cloud cover, and other factors. Therefore, we also calculated ‘lunar-minutes’ for each night in the study as the product of moon illumination (0–1.0) and the time (in minutes) the moon was above the horizon after the end of civil twilight and before the onset of twilight the following morning (moonrise, moonset, and beginning and ending on twilight obtained from [33]). Qualitatively, our results were the same whether the effects on moonlight on GUD were analyzed using the fraction of moon illuminated (0–1.0) or lunar-minutes, and they were strongly positively correlated (*r* = 0.9). For brevity, here we present results only for analyses using lunar-minutes. Kotler et al. [27] showed that the sequence and phase of the lunar cycle is also important, with rodents responding differently to waxing and waning moons with the same amount of illumination. In our study, locations 1 and 3 were run with waning moons, while location 2 was run with a waxing moon.

We measured plant cover at each feeding station by laying down a 1m^2^ frame centered on each food station and visually estimating the cover (to nearest 10%) of each plant species with at least 10% cover. To aid in visualization, the frame was demarked with intersecting lines to demark 25 equally sized (20 × 20 cm) areas. Cover data were not normally distributed, so we analyzed differences in cover between habitats using non-parametric Kruskal-Wallis tests. We performed tests for each of the 24 species with at least 10% cover at feeding station, as well as for total cover, so we used Bonferroni’s adjustment to control for experimental wise error rate by lowering alpha for these tests down to 0.002.

We also placed motion-activated infrared video cameras (Primos Truthcam35) at some of the feeding stations to confirm the presence of rodent seed predators. Locations for cameras were chosen to minimize risk of theft, so they are not random sample of food patches and are used here only for descriptive purposes. Four cameras were deployed on each of the pair of sites run simultaneously. The cameras were attached at a height of 1 m to wooden stakes driven into the sand approximately 1.5 m away from a feeding station and oriented downward toward the interior of the station’s box. After trial runs, we learned that at this close range the infrared light source needed to be dampened to avoid overexposing the video, which we accomplished by using black electrical tape to obscure some of the bulbs.

To examine the GUD data, we first performed a Mantel test on the geographic distance and the mean GUD among feeding stations in order to assess the appropriateness of treating GUDs from each station as spatially independent samples. Independence was confirmed (see Results), so we treated each feeding station independently in subsequent analyses. GUDs were normally distributed, so we used parametric analyses to maximize statistical power. We analyzed the data in two ways. First, we calculated the mean number of lunar-minutes over each 6 night trial, yielding three values (one for each location [pair of grids run simultaneously]). We used the mean GUD at each feeding station over the 6-night trial as the response variable in a 2-way ANOVA with habitat (*Ammophila*-dominated or restored) as one factor and mean lunar-minutes as the other factor. This analysis assessed main and interactive effects of habitat and lunar-minutes, but averaging moonlight over six nights masked what could be important variation. So, second, we used the GUD at each feeding station each night as the response variable in a linear mixed effects model with each station as a subject, night (1–6) nested within location as a subject factor (repeated measures), habitat as a fixed between subject effect, number of lunar-minutes as a fixed covariate. Over a six-night trial, the number of lunar minutes changed while the location stayed constant, so location was nested within lunar minutes as a random effect. We used an alpha level of 0.05 for these analyses, which were performed in R [34]. This study was carried out in accordance with Federal animal welfare guidelines. The protocol was approved by Humboldt State University’s Institutional Animal Care and Use Committee (IACUC permit 10/11.W.63.A).

## Results

Total plant cover was significantly higher in the *Ammophila*-dominated habitat (92.3%) than in the restored habitat (44.9%; Table 1). The *Ammophila*-dominated habitat was comprised almost exclusively of *Ammophila* (85.6% cover); the species with the next highest cover was *Baccharus pilularis* (2.7% cover). The restored habitat was much more diverse, with five species showing significantly higher cover than in the *Ammophila*-dominated habitat: *Abronia latifolia*, *Achillea millefolium*, *Erigonum latifolium*, *Lathyrus littoralis*, and *Solidago spathulata* (Table 1). Overall, 24 species were detected with at least 10% cover at a feeding station, 10 species in the *Ammophila*-dominated habitat, 18 in the restored habitat, and four species that occurred in both. Four of the species that are considered invasive: *Ammophila*, *Briza maxima*, and *Lupinus arboreus* were recorded only in the *Ammophila*-dominated habitat, while *Carpobrutus edulis* was recorded only in the restored habitat.

**Table 1:** Plant species detected (with 10% on at least one sample point) on *Ammophila*-dominated and restored habitats in northwestern California, June-August 2011 (*n* = 75 sample 1m^2^ sample frames in each habitat).

	*Ammophila*-dominated		Restored			
Species	Mean	Range	Mean	Range	H	P
***Abronia latifolia***	0	0	2.4	0–30	11.1	< **0.001**
***Achillea millefolium***	0	0	5.9	0–30	35.3	< **0.001**
*Ambrosia chamissonis*	0.4	0–10	1.2	0–20	1.8	0.183
***Ammophila arenaria*** *	85.6	50–100	0	0	128.8	< **0.001**
*Artemesia pynocephala*	0	0	0.5	0–10	4.1	0.043
***Baccharus pilularis***	2.7	0–30	0	0	16.5	< **0.001**
*Briza maxima* *	0.5	0–10	0	0	4.1	0.043
*Cakile maritime*	0	0	0.5	0–20	3.0	0.081
*Calystegia soldanella*	0	0	0.5	0–10	4.1	0.043
*Camissonia cheiranthifolia*	0	0	0.4	0–10	3.0	0.081
*Carpobrutus edulis* *	0	0	0.5	0–10	4.1	0.043
*Erigeron glaucus*	0	0	0.1	0–10	0.1	0.317
***Erigonum latifolium***	0.1	0–10	10.4	0–50	52.7	< **0.001**
*Fragaria chiloensis*	0	0	1.9	0–30	9.5	0.002
*Juncus breweri*	0.3	0–10	0	0	2.0	0.156
***Lathyrus littoralis***	0	0	7.1	0–40	35.3	< **0.001**
*Layia carnosa*	0	0	0.1	0–10	0.1	0.317
*Leymus mollis*	0	0	0.7	0–10	5.1	0.023
*Lonicera involucrate*	0.1	0–10	0	0	0.1	0.317
*Lupinus arboreus* *	0.1	0–10	0	0	0.1	0.317
*Rubus ursinus*	0.8	0–10	0.4	0–30	3.6	0.058
***Solidago spathulata***	1.3	0–20	10.5	0–30	47.6	< **0.001**
*Tanacetum camphoratum*	0	0	0.1	0–10	0.1	0.317
*Umbellata breviflora*	0	0	1.6	0–30	8.4	0.004
**Total Cover**	92.3	70–100	44.9	10–80	113.2	< **0.001**

Bold font indicates a significant difference between habitats based on Kruskal-Wallis tests with alpha value lowered to 0.002 to adjust for experiment-wise error rate.

* indicates non-native invasive species.

Results of the Mantel tests indicated that there was little evidence for spatial autocorrelation among the feeding stations. Only one of the six tests (one test for each grid) was significant (*P* = 0.014 based on 9999 permutations), and with Bonferroni adjustments for multiple comparisons (alpha lowered to 0.008), none was significant. Therefore, we analyzed the data with each GUD station (25/grid, 6 grids) treated as a sample unit.

The number of lunar-minutes during a night varied from 28.1 to 281.4, with moon illumination varying from 0.23 to 0.83, over the course of the study. The mean number of lunar-minutes (±1 SE) was 176.8 (±30.3) for location 1, 116.4 (±31.5) for location 2, and 155.0 (±33.1) for location 3 (2 simultaneous grids at each location); these differences were statistically significant (ANOVA: F_2,897_ = 55.8, *P* < 0.001). Thus, these locations experienced high, low, and intermediate values of nocturnal moonlight during our GUD trials.

**Fig 2:**
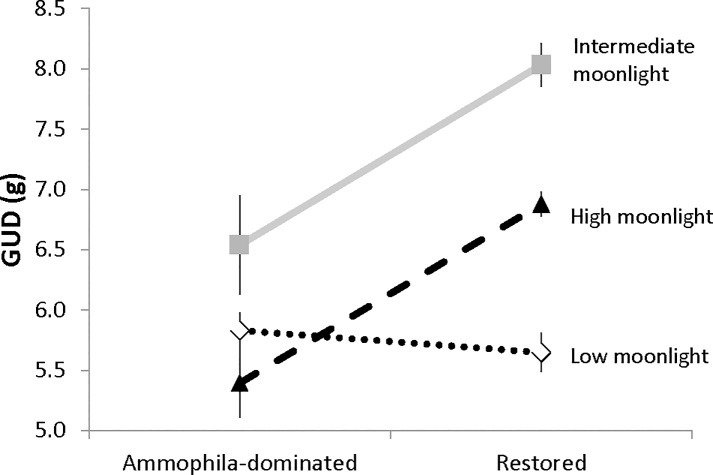
Effect of habitat and moonlight on giving up density. Mean (±1 SE) nocturnal giving-up-density of food (GUD) left behind on feeding stations deployed in *Ammophila*-dominated and restored habitats in the coastal dunes of Northwestern California, June-July 2011. The experiment was run under three moonlight conditions based on the number of lunar-minutes (product of fractional moon illumination and the number of minutes the moon was above the horizon after the end of civil twilight and before the onset of twilight the following morning): low moonlight (116.4), intermediate moonlight (155.0), and high moonlight (176.8).

A two-way ANOVA revealed strong effects of habitat, lunar-minutes, and their interaction on GUD (Table 2). Overall, there was a significant main effect of habitat, with higher GUDs in the restored than in the *Ammophila*-dominated habitat, consistent with the hypothesis that perceived predation risk diminishes with abundant cover. The main effect of lunar-minutes was also significant, with GUDs highest at intermediate values and the lowest GUDs on nights with low lunar-minutes. There also was a strongly significant interaction between habitat and lunar-minutes; GUD was lower in *Ammophila* (lower predation risk) only when the number of lunar minutes was intermediate or high (Fig. 2).

**Table 2:** ANOVA table for 2-way analysis of habitat, moonlight, and their interaction on nocturnal GUD in *Ammophila*-dominated and restored habitats in northwestern California, June-August 2011.

Source	Num. df	Denom. df	F	P
Habitat	1	149	28.4	< 0.001
Lunar-minutes	2	149	58.9	< 0.001
Habitat*lunar-minutes	2	149	28.6	< 0.001

There were 25 feeding station on each of 6 grids (3 locations with 2 grids each), run for 6 nights each. Analyses were run on the mean GUD and mean number of lunar-minutes over 6-night trial on each grid.

The mixed effects linear model also revealed main effects of habitat and lunar-minutes, as well as location (Table 3). GUDs were higher in restored than *Ammophila*-dominated habitat. There was also a main effect of location, with location 3 having higher GUD than the others, and a significant interaction between lunar-minutes and location, with GUDs at location 3 (intermediate lunar minutes) showing a strong positive association with increasing lunar minutes, whereas there was a slightly negative association with increasing lunar-minutes elsewhere. However, the interaction between habitat and moonlight was not statistically significant. Effects on individual nights within the six = night trial were not discernible because they were treated as random effects. In a few cases GUD values exceeded 10 g (initial seed weight) due to little or no foraging combined with persistent moisture or imperfect removal of sand, but this capacity should not have differed significantly between habitats or over lunar-minutes. Remote video evidence confirmed that seeds in the foraging trays were foraged for and consumed by (at least): California voles (*Microtus californicus*) and deer mice (*Peromyscus maniculatus*), which we observed in each of the two habitats. Other species observed also included gray foxes (*Urocyon cinereoargenteus*) and striped skunks (*Mephitis mephitis*), but they did not forage on seeds in the trays.

**Table 3:** ANOVA table for mixed effects model examining effects of habitat, moonlight, and their interaction on GUD in *Ammophila*-dominated and restored habitats in northwestern California, June-August 2011.

Source	Coefficient	SE	df	t	P
**Intercept**	**9.403**	**1.295**	**880**	**7.261**	<**0.001**
**Treatment (restored)**	**0.682**	**0.304**	**880**	**2.247**	**0.025**
Location 2	-1.053	1.568	7	-0.671	0.523
Location 3	3.085	1.661	7	1.857	0.106
**Lunar-minutes**	**-0.021**	**0.007**	**7**	**-3.069**	**0.018**
Treatment×lunar-minutes	0.001	0.002	880	0.695	0.487
Location 2×lunar-minutes	-0.004	0.009	7	-0.440	0.673
**Location 3**×**lunar-minutes**	**0.024**	**0.009**	**7**	**2.672**	**0.031**

There were 25 feeding stations on each of 6 grids (3 locations with 2 grids each), run for 6 nights each. Analyses were run with each station as a subject, night (1–6) nested as a random within subject factor (repeated measures), habitat as a fixed between subject effect, the number of lunar-minutes as a fixed covariate, and location nested within lunar-minutes as a random effect. Effects on individual nights within the six = night trial are not reported because they were treated as random effects. Bold font indicates statistically significant effects.

## Discussion

Results of this study support the hypothesis that cover provided by the invasive beach grass *Ammophila arenaria* lowers the perceived predation risk for foraging rodents. Overall, the amount of seeds left behind by foraging rodents (the GUD), an indicator of perceived predation risk [24], was lower in the thick uniform vegetation on *Ammophila*-dominated habitat than it was in the more sparsely and diversely vegetated restored habitat. There was also some support, though less consistent, for the hypothesis that the perceived safety provided by cover may depend on moonlight, with strongest differences during nights with more lunar light. Both a 2-way ANOVA and a mixed model showed significant main effects of lunar-minutes on GUD, with the former also showing a strong interaction in which GUD was lower in *Ammophila* only when the number of lunar minutes was intermediate or high (Fig. 2). This interaction was not significant in the mixed model, which did not pool results across the six nights of each trial.

Our findings illustrate that foraging rodents, well known to be risk-averse during moonlit nights [35], [12], are also affected by the cover from an invasive plant [8, 36]. This result is important because it has implications for granivory and perhaps plant demography in invaded and restored coastal habitats. In central coastal California, Maron and Simms [19], [20] found that rodents limit the establishment and recruitment of *Lupinus arboreus*, a native shrub in their study area. *Lupinus arboreus* is a large, N-fixing shrub, and it influences soil nutrients [37]. In our study region of northwestern California, *Lupinus arboreus* is a non-native invader [38], so understanding if and how native rodents may be able to help slow its spread—possibly aiding restoration efforts—is a high priority for future research. However, high rates of granivory in habitats with *Ammophila* could also affect native plants—possibly inhibiting restoration. Dangremond et al. [21] found that rodent granivory of an endangered plant, *Lupinus tidestromii*, was higher near *Ammophila*-dominated habitat in coastal dunes of central California, creating apparent competition between *Ammophila* and native plants. Indeed, future research in this system should work toward unraveling the complex trophic links formed by a non-native invasive plant (i.e., *Ammophila*) providing cover favored by native rodents, which likely forage on and potentially limit the recruitment of native and non-native plants, some of which have ecosystem consequences of their own (i.e., *Lupinus arboreus*). The ongoing removal of *Ammophila* in our study region may create unique opportunities for experimental studies of how cover affects rodents and their implications for the overall dune ecology.

Mattos and Orrock [8] also found that an invasive plant, in their case *Lonicera maackii* in oak-hickory forests of Missouri USA, lowered risk perceived by granivorous rodents, but that this effect was contingent on abiotic factors, including both moonlight and temperature. Our results extend their finding to another invasive species and region, suggesting that moonlight may more generally mediate the effect of safety perceived in habitats dominated by high densities of invasive plants. Temperature did not vary enough among nights in our study period (June/July 2010) to merit analysis, so we cannot comment on possible effects of temperature in our system. Together, results of our study and those of Mattos and Orrock [8] underscore that simply examining the effects of invasive plants on wildlife behavior may not be enough, because their effects may be altered by abiotic factors.

Kotler et al. [27] illustrated the importance of the lunar cycle to rodent foraging. They clarified that is not merely a matter of light intensity, but also a matter of sequence and context [27]. Nocturnal rodents forage and cache food during the darker period of the cycle, building up their energetic state. On the brighter period of the cycle, they lower predation risks by foraging less and relying on their reserves that deplete during this period. Therefore, as the moon wanes and the nights become darker, rodents invest more effort in foraging to recover their state effort (which should lead to low GUDs), whereas as the moon waxes and the light increases they reduce foraging (which should raise GUDs). In our study, the sites with high and intermediate moonlight (sites 1 and 3) were monitored during a waning moon when rodents may increase foraging to recover state, while the site with low moonlight (site 2) was monitored during the time when rodents reduce foraging. Therefore, due to the timing of the data collection, it is impossible to determine whether rodents foraged more at invaded sites on locations 1 and 3 because of the number of lunar minutes, the moon phase, or both.

The giving up density of food in a foraging patch is a balance of the benefit (i.e., the food harvest rate) and three costs: the costs of handling and metabolizing the food, the cost of missed opportunities of foraging elsewhere, and the costs of predation risk [7]. We believe that the differences we measured in GUD between habitats reflects variation in perceived predation risk because habitat differences in the other costs were likely negligible. We used the same food (shelled sunflower seeds) and sand for all foraging stations, so handling and metabolic costs should have been consistent. Sunflower seeds are energetically very dense, with 560 cal per 100 g of seeds [39], and laboratory trials consistently show they are highly desirable for granivorous rodents and in many cases preferred over wild native seeds (e.g., [39], [40], [41]). Thus, the sunflower seeds should have diminished and dampened variation in the cost of missed opportunity outside the foraging trays. Also, during our study period (mid-late summer), few plant species have mature seeds in either habitat, and wild foods are relatively rare. Those that are present include the previous year’s *Ammophila* crop. Although we did not measure differences in standing crop of wild seeds between habitats, if any difference existed it would almost certainly be that wild seeds were more available in the *Ammophila*-dominated habitat. If so, this would be a conservative bias, since a higher missed-opportunity cost stemming from more abundant wild foods would raise the GUD in *Ammophila*—the opposite of our observed effect of a significantly lower GUD in *Ammophila*.

Perceived predation may be revealed by measuring GUDs, but this may or may not reflect actual risk of predation. Several studies involving GUDs suggest that rodents may perceive predation risk indirectly by monitoring vegetative structure and responding accordingly, regardless of other direct cues (e.g., number of predators) or true risk of death [42], [43]. For example, Orrock et al. [12] found that mice at the Savannah River site in North Carolina perceived predation risk as a function of vegetative cover and moon illumination and were unresponsive to experimental direct cues (urine from predators). In our study system, the most important predators of rodents are probably gray foxes (*Urocyon cinereoargenteus*), striped skunks (*Mephitis mephitis*), feral cats *(Felis catus)*, barn owls (*Tyto alba*), and perhaps some snakes (e.g., California red-sided garter snake *Thamnophis sirtalis infernalis*). All of these predators except the snakes should be hindered by the cover [36]; the snakes should find it an improved habitat for ambush sites. Future research should examine the prevalence and activity of predators in *Ammophila*-dominated and restored habitats in these coastal dunes.
